# Durable Clinical Benefit in Patients with Advanced Cutaneous Melanoma after Discontinuation of Anti-PD-1 Therapies Due to Immune-Related Adverse Events

**DOI:** 10.1155/2019/1856594

**Published:** 2019-07-25

**Authors:** Umang Swami, Varun Monga, Aaron D. Bossler, Yousef Zakharia, Mohammed Milhem

**Affiliations:** ^1^Division of Oncology, Department of Internal Medicine, Huntsman Cancer Institute, University of Utah, Salt Lake City, UT, USA; ^2^Division of Hematology, Oncology and Blood & Marrow Transplantation, University of Iowa Hospitals and Clinics, 200 Hawkins Dr., Iowa City, IA 52242, USA; ^3^Department of Pathology, University of Iowa Hospitals and Clinics, 200 Hawkins Dr., Iowa City, IA 52242, USA

## Abstract

**Introduction:**

Anti-PD-1 therapies, pembrolizumab and nivolumab, are currently the standard of care for treatment of patients with metastatic melanoma. Treatment is usually continued until toxicity or disease progression. Though these therapies are well tolerated, some patients discontinue them due to immune-related adverse events (irAE). Discontinuation of therapy brings challenges to their management due to limited treatment options and lack of long-term prognostic information for these patients. Herein, we reviewed patients at our institution to analyze their clinical outcomes.

**Materials and Methods:**

Charts of 1264 consecutive patients enrolled between 8/1/2012 and 7/31/2017 at Melanoma Skin & Ocular Tissue Repositories at Holden Comprehensive Cancer Center at the University of Iowa Hospitals and Clinic were reviewed. Eligible patients were those who received single-agent anti-PD-1 therapy and subsequently discontinued it due to irAE. Reviewed data included patient demographics, prior medical history, baseline disease parameters, and outcomes. Kaplan-Meier survival analysis was done to determine progression-free survival (PFS) and overall survival (OS).

**Results:**

Overall 169 patients with advanced, unresectable, or metastatic cutaneous melanoma received anti-PD-1 therapy of which 16 (9.5%) white, non-Hispanic patients with median age of 64.5 (range 35 to 81 years) discontinued treatment due to irAE. Fifteen patients received pembrolizumab and one received nivolumab. The median duration of treatment was 4.7 (range 0.7 to 11.5) months. Median follow-up was 30.3 (range 4.6 to 49.4) months. Median PFS was 24.6 months and median OS was not reached. Durable clinical benefit (time to progression or next treatment of more than 6 months from last treatment) was observed in 13 (81.2%) patients. At the time of analysis, 8 patients had progressed and 4 patients died (all-cause).

**Discussion:**

Our results suggest that advanced melanoma patients discontinuing anti-PD-1 therapy due to irAE usually experience durable clinical benefit. However, caution is needed with these agents in patients with underlying autoimmune diseases.

## 1. Introduction

Monoclonal antibodies targeting programmed cell death 1 protein (PD-1) have shown to improve progression-free survival (PFS) and overall survival (OS) in patients with metastatic melanoma [1]. The advent of anti-PD-1 antibodies along with antibodies targeting cytotoxic T-lymphocyte-associated protein 4 (CTLA-4) and therapies targeting BRAF mutation has provided multiple options to treat patients with metastatic melanoma. Due to these therapies, the median overall survival of metastatic melanoma has improved from 6 months to more than 3 years [2–4]. Currently, two monoclonal antibodies targeted against PD-1 have been approved as first-line agents for the treatment of metastatic melanoma [1].

PD-1 inhibitors can lead to durable responses [1, 5] and have better toxicity profiles as compared to CTLA-4 inhibitors and targeted therapies [1, 3, 4]. However, approximately, 86% of patients experiencing treatment-related toxicities (all grades) and severe (grade 3 or higher) toxicities are in the range of 17 to 22% [3, 4]. Treatment discontinuation due to immune-related adverse events (irAEs) is estimated to occur in 15% to 25% of patients [3, 4]. These patients lack effective therapies as many of them do not have actionable mutation, and even in patients with BRAF mutation, the median PFS with BRAF-MEK inhibitors is low (11 to 15 months) with a high rate of toxicities [1, 6, 7]. Therefore, there is a need to understand the long-term prognosis of patients who undergo treatment discontinuation due to irAE to guide management decisions.

## 2. Materials and Methods

After approval from the Institutional Review Board, data of 1264 patients enrolled at Melanoma Skin & Ocular Tissue Repositories at Holden Comprehensive Cancer Center at the University of Iowa Hospitals and Clinics from 8/1/2012 to 7/31/2017 was reviewed. Patients with unresectable, advanced, or metastatic cutaneous melanomas who discontinued anti-PD-1 therapies due to irAEs were identified and their charts were reviewed in detail. Reviewed data included demographics (gender, race, and ethnicity), mutational status, prior treatment regimens including radiation therapy, melanoma metastases to brain and liver, and irAEs. Identified patients were followed till 02/26/2019. Progression (clinical or radiological) and responses were determined by iRECIST [8] and clinic notes. Outcomes with anti-PD-1 therapies including PFS, time from treatment discontinuation to progression, and OS were collected. Common Terminology Criteria for Adverse Events Criteria Version 4.03 were used to grade irAE [9].

### 2.1. Statistical Analysis

Baseline clinical and disease characteristics were summarized as medians and ranges for continuous variables and as numbers and percentages for categorical variables. Kaplan-Meier survival analysis was used to determine PFS and OS. Time was calculated from initiation of anti-PD-1 treatment to progression or, new treatment for PFS, time from last treatment to next treatment or progression for clinical benefit and to death due to any cause for OS. Durable clinical benefit was defined as time of 6 months or more to progression or next treatment from last therapy. Survival curves were drawn using GraphPad Prism Version 7.04 (GraphPad Software, Inc., San Diego, CA).

## 3. Results

### 3.1. Baseline Characteristics

Overall 169 patients with advanced, unresectable, or metastatic cutaneous melanoma received anti-PD-1 therapy, of which 16 (9.5%) patients discontinued treatment due to irAEs. All patients who discontinued treatment were white and non-Hispanic. The median age was 64.5 (range 35 to 81) years. Ten (62.5%) patients were male and six (37.5%) were female. Eight (50%) patients had a BRAF mutation. Four (25%) patients had brain metastasis and one (6.3%) had liver metastasis. Six (37.5%) patients had prior treatment with ipilimumab for metastatic disease.  Table 1 provides the baseline demographics and clinical profile of all patients.

### 3.2. Treatment

Fifteen (93.7%) patients received pembrolizumab and one (6.3%) patient received nivolumab. Eight (50%) received anti-PD-1 therapy as the first line, seven (43.7%) as second, and one (7.3%) as the third line. Patient 6 received bevacizumab in addition to pembrolizumab after 6 cycles of single-agent pembrolizumab due to pseudoprogression in the brain. None of the patients received concomitant radiation with anti-PD-1 therapy. The median duration of treatment was 4.7 (range 0.7 to 11.5) months.  Table 2 provides a summary of treatment duration, line of therapy, and outcomes of each patient.

### 3.3. irAEs

The median duration from initiation of treatment to development of irAE was 4 (range 0.5-11.5) months. Most commonly observed toxicities leading to treatment discontinuation included diarrhea and rash (4/16, 25% each) and arthritis (3/16, 18.7%). Other observed toxicities included colitis (2), neuropathy (2), pancreatitis (2), fatigue, nausea, nephritis, adrenal insufficiency, hypothyroidism, low mood, mouth sores, hepatitis, uveitis, and myasthenia flare (1 each). Ten (62.5%) patients experienced grade 3 or higher toxicities.  Table 3 provides a summary of these toxicities and immunosuppressive agents used for their management. Apart from the mentioned toxicities, patient 1 developed grade 2 hypothyroidism on day 81 and patient 8 developed grade 2 pityriasis lichenoides after 2 cycles secondary to pembrolizumab. However, both did not lead to treatment discontinuation.

### 3.4. Outcomes

**Figure 1:**
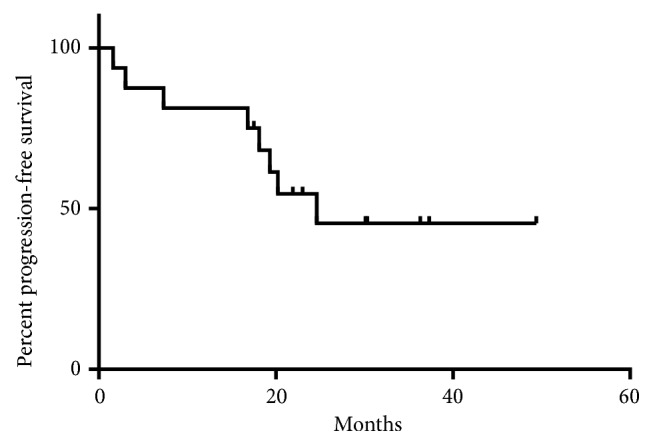
Kaplan-Meier curve for progression-free survival.

**Figure 2:**
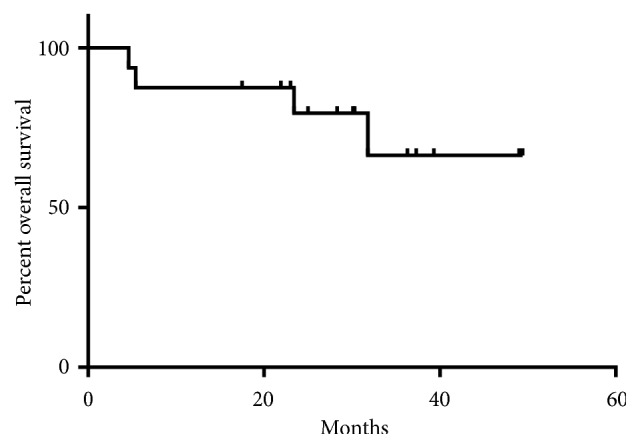
Kaplan-Meier curve for overall survival.

Median follow-up was 30.3 (range 4.6 to 49.4) months. Eight (50%) had complete response, five (31.2%) had partial response, two (12.5%) had stable disease, and one (6.3%) had progressive disease as best response to treatment. At the time of analysis, 8 patients had progressed and 4 patients experienced all-cause mortality, of which one death was unrelated to melanoma. Median PFS was 24.6 months and median OS was not reached due to durable disease control (Figures 1 and 2). Durable clinical benefit was observed in 13 (81.2%) patients.

**Figure 3:**
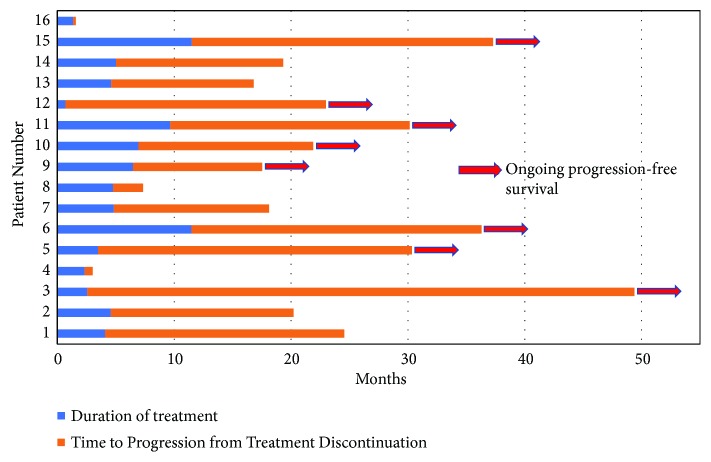
Swimmer's plot demonstrating duration of treatment and progression-free survival.

Patients number 4 and 16 had a PFS of less than 6 months PFS (Figure 3). Patient 4 had underlying thymoma and patient 16 had myasthenia gravis. With pembrolizumab, they experienced severe neuroimmune toxicity and flare-up of myasthenia gravis, respectively, which caused rapid clinical deterioration. All patients except one received steroids (oral, topical, or ophthalmic), while two received steroid-sparing agents. Of the eight patients who progressed, three were retreated with pembrolizumab-containing regimen. Of these three patients, one developed pembrolizumab induced psoriasis, while the remaining two tolerated it without any significant side effects (Table 2).

## 4. Discussion

Prior studies have given conflicting evidence with regard to the association of PD-1-related irAEs with survival outcomes in melanoma patients. Freeman-Keller et al. reported OS association with rash and vitiligo, while no survival benefit was seen with other irAEs including endocrinopathies, colitis, or pneumonitis. In the study, 12-week landmark PFS analysis was difficult to interpret due to exclusion of a large number of patients [10]. Another pooled analysis of 576 patients by Weber et al. reported that after exclusion of patients progressing before 12 weeks, there was no difference in PFS between patients without AEs and those with one to two AEs or between those with any-grade AE and all patients [11]. In another study by Indini et al., irAE was associated with improved PFS [HR 0.47 (95% CI 0.26, 0.86); p = 0.016] and OS [HR 0.39 (95% CI 0.18, 0.81); p = 0.007] on multivariable analysis in patients who received more than 2 doses of anti-PD-1 therapies [12]. Quach et al. reviewed single institution data of 318 patients treated with anti-PD-1 therapies with or without ipilimumab and reported a better response rate (60.0% vs. 28.6%; *χ*^2^*p* < .001), median PFS (797 vs. 112 days; log rank p < .001), and median OS (1691 vs. 526 days; p< .001) in patients who experienced cutaneous side effects. Superior outcomes with regard to response rate, PFS, and OS were seen with vitiligo and rash as compared to pruritus [13].

In this study, we analyzed the impact on PFS and OS for patients who discontinued anti-PD-1 therapy due to irAEs. Complete response was seen in patients with irAEs from a wide spectrum and grade of toxicities including grade 2 and 3 diarrhea/colitis (2 patients), grade 3 rash (3 patients), grade 3 pancreatitis (1 patient), grade 2 nephritis (1 patient), and grade 2 hypothyroidism, low mood, mouth sores, and rash (1 patient). We found a durable clinical benefit in thirteen patients (81.2%) discontinuing PD-1 directed therapy due to irAEs; none of them progressed in more than one year and 6 (37.5%) in more than 2 years (Figure 3). The benefit was not seen in patients with an underlying autoimmune disease like myasthenia gravis or who are at a higher risk of developing it, like patient 4 with thymoma [14]. However, because of the small sample size, no definitive conclusion should be drawn; this finding needs to be explored further.

KEYNOTE-001 has reported durable complete remission after discontinuation of pembrolizumab in patients with complete remission [5]. However, we found durable benefit in patients with residual disease also. Though the mechanisms for this phenomenon are not clear, it is very much possible that PD-1 blockade may result in an adaptive memory immune response providing antitumor effect even after treatment cessation and translating as irAEs [15–17]. A similar study evaluated 19 patients with metastatic renal cell carcinoma who experienced an initial clinical response but after irAE discontinued PD-1/PD-L1 therapy. The median time on PD-1/PD-L1 therapy was 5.5 months; median TTP was 18.4 months and durable clinical benefit off treatment (TTP > 6 months) was observed in 68.4% (n=13) patients [15].

Pollack et al. reported that metastatic melanoma patients who discontinued CTLA4/PD-1 blockade due to irAEs can be rechallenged with anti-PD-1 therapies. The study showed a relatively higher rate of recurrent or different irAEs on resumption of anti-PD-1 therapies. They concluded that this approach can be used in selected patients [18]. We also found that patients who had to discontinue pembrolizumab due to irAE were able to be treated again with pembrolizumab-based therapies with manageable toxicities in two of the three patients. Our study has similar limitations as most retrospective studies including selection bias, chances of errors during data entry and confounding.

In summary, we present outcomes of 16 patients with metastatic melanoma who discontinued anti-PD-1 therapies due to immune toxicities. To the best of our knowledge, it is the largest series to date of real-world patients. Its strength includes a long-term follow-up and comprehensive analysis of each case which can help generate multiple hypotheses in combination with other relevant studies. Our results show durable clinical benefit in patients who discontinue anti-PD-1 therapies after irAEs. However, this needs to be confirmed in larger cohorts. We also need more comprehensive preclinical and clinical studies to determine how individual patient variables, cancer and immune system, interact to cause irAEs in only a select few patients while sparing a majority. We also need to develop predictive and prognostic novel biomarkers for anti-PD-1 therapies and also for irAEs.

## Figures and Tables

**Table 1:** Patient demographics and clinical profile at the time of starting anti-PD-1 therapy.

Pt. No.	Age (years)	Gender	Mutational status	Brain metastasis	Liver metastasis	Prior adjuvant therapy	Prior therapy for metastatic disease	Prior immune-related adverse event	Radiation therapy within prior 3 months	Prior relevant history or autoimmune disease
1	63	Male	BRAF V600E	Yes	No	Interferon	Ipilimumab, stereotactic radiosurgery	Colitis	Yes	None

2	61	Female	BRAF Negative	No	No	Radiation	Ipilimumab	Colitis, rash	No	Lichen planopilaris

3	72	Female	BRAF Negative	No	No	GM-CSF	Ipilimumab	Colitis	No	None

4	81	Male	BRAF V600K, GNAQ, RAC1, POLD1, TERT	Yes	No	No	Radiation, craniotomy and tumor resection, stereotactic radiosurgery	NA	Yes	Thymoma

5	64	Male	BRAF V600E	No	No	Radiation	Ipilimumab with talimogene laherparepvec	None	No	None

6	59	Female	NRAS	Yes	No	Interferon	Ipilimumab, craniotomy and tumor resection, radiation	None	Yes	None

7	66	Female	BRAF V600E	No	No	No	NA	NA	No	None

8	35	Male	BRAF V600E, PTEN, MET	No	No	Interferon	Ipilimumab with interleukin-2, ipilimumab	Diarrhea, nausea, vomiting, hyperbilirubinemia, acute kidney injury, oliguria, tachycardia	No	None

9	61	Male	TP53	No	No	No	NA	NA	No	None

10	77	Male	KIT p.W557G	Yes	No	Radiation	Stereotactic radiosurgery	NA	Yes	None

11	65	Male	NRAS, TP53, KIT, SF3B1, CDK2NA	No	No	No	NA	NA	No	None

12	67	Female	Not done	No	No	No	NA	NA	No	None

13	78	Male	BRAF p.L597S	No	No	No	Carboplatin with paclitaxel	NA	No	No

14	64	Male	BRAF V600K	No	No	Radiation, ipilimumab	NA	Rash	No	No

15	47	Female	BRAF V600E	No	No	Interferon	Vemurafenib with high dose interleukin-2, vemurafenib with decitabine, craniotomy, stereotactic radiosurgery	Rash, diarrhea	Yes	No

16	75	Male	BRAF negative	No	Yes	No	NA	NA	No	Myasthenia gravis

**Table 2:** Details of the line of anti-PD-1 therapy, duration of treatment, survival, and subsequent treatment on progression.

PN	Anti-PD-1 Therapy	Line of anti-PD-1 therapy	ECOG PS	Response	DOT (months)	PFS (months)	TPTD (months)	Overall Survival (months)	Subsequent therapy
1	Pembrolizumab	second	1	PR	4.1	24.6	20.5	39.3*∗*	Radiation, pembrolizumab with development of pembrolizumab induced psoriasis

2	Pembrolizumab	second	0	CR	4.6	20.2	15.6	49.0*∗*	Radiation, pembrolizumab with TLR9 agonist without immune toxicities

3	Nivolumab	second	1	CR	2.6	49.4^+^	46.8^+^	49.4*∗*	NA

4	Pembrolizumab	first	1	SD	2.3	3.0	0.7	4.6	Dabrafenib with trametinib

5	Pembrolizumab	second	0	PR	3.5	30.3^+^	26.9^+^	30.3*∗*	NA

6	Pembrolizumab	second	1	CR	11.5	36.3^+^	24.8^+^	36.3*∗*	NA

7	Pembrolizumab	first	1	PR	4.8	18.1	13.3	23.4	Radiation

8	Pembrolizumab	second	1	SD	4.8	7.3	2.6	28.3*∗*	Craniotomy and surgical resection, stereotactic radiosurgery, vemurafenib with cobimetinib

9	Pembrolizumab	first	0	CR	6.5	17.5^+^	11.0^+^	17.5*∗*	NA

10	Pembrolizumab	first	1	CR	6.9	21.9^+^	15.0^+^	21.9*∗*	NA

11	Pembrolizumab	first	0	CR	9.7	30.1^+^	20.5^+^	30.1*∗*	NA

12	Pembrolizumab	first	0	CR	0.7	23.0^+^	22.3^+^	23.0*∗*	NA

13	Pembrolizumab	second	2	PR	4.6	16.8	12.2	25.0*∗*	Radiation, dabrafenib with trametinib

14	Pembrolizumab	first	0	PR	5.0	19.3	14.3	31.8	Pembrolizumab with TLR9 agonist without immune toxicity, radiation, vemurafenib with cobimetinib

15	Pembrolizumab	third	1	CR	11.5	37.3^+^	25.8^+^	37.3*∗*	NA

16	Pembrolizumab	first	0	PD	1.3	1.6	0.2	5.4	Temozolomide

PN: patient number. ECOG PS: Eastern Cooperative Oncology Group Performance Status.

PR: partial response. CR: complete response. *∗*Alive.

SD: stable disease. PD: progressive disease. ^+-^Censored, no evidence of progression.

DOT: Duration of treatment.

TPTD: time to progression from treatment discontinuation.

**Table 3:** Details of immune-related adverse events leading to discontinuation of anti-PD-1 therapy and their treatment.

Patient Number	irAE leading to discontinuation of anti-PD-1 therapy	Time of first presentation of any grade irAE from initiation of therapy (months)	Immune suppressive agents for treatment of irAE
1	Grade 2 inflammatory arthritis and neuropathy	4.1	Prednisone

2	Grade 2 diarrhea	4	Budesonide

3	Grade 3 colitis and diarrhea	2.6	Prednisone, budesonide
	Grade 2 adrenal insufficiency	4.1	Hydrocortisone

4	Grade 3 sensorimotor polyneuropathy	2.3	Prednisone

5	Grade 1 diarrhea	2.8	Budesonide
	Grade 3 pancreatitis and colitis	3.5	Budesonide

6	Grade 3 rash	11.5	Dexamethasone, topical steroids

7	Grade 2 inflammatory arthritis	4.6	Prednisone, methotrexate

8	Grade 2 fatigue, nausea, diarrhea, arthritis	4.8	None

9	Grade 3 pancreatitis	6.5	Prednisone

10	Grade 2 nephritis	6.9	Prednisone

11	Grade 2 hypothyroidism, low mood, mouth sores, rash	9.7	Topical steroids

12	Grade 3 rash	0.5	Prednisone

13	Grade 3 hepatitis	2.1	Prednisone, budesonide

14	Grade 3 uveitis	3.9	Prednisone, ophthalmic prednisolone

15	Grade 3 rash	11.3	Prednisone, topical steroids

16	Grade 4 myasthenia flare	0.8	Plasma exchange, mycophenolate mofetil, prednisone, intravenous immunoglobulin, abatacept

IrAE: immune-related adverse events.

## Data Availability

The data used to support the findings of this study are available from the corresponding author upon request.

## Abbreviations

PD-1: Programmed cell death 1 protein
PFS: Progression-free survival
OS: Overall survival
CTLA-4: Cytotoxic T-lymphocyte-associated protein 4
irAE: Immune-related adverse events
TTP: Time to progression.
